# Commensal oral microbiota impacts ulcerative oral mucositis clinical course in allogeneic stem cell transplant recipients

**DOI:** 10.1038/s41598-022-21775-3

**Published:** 2022-10-20

**Authors:** Julia S. Bruno, Vitor Heidrich, Franciele H. Knebel, Vinícius Campos de Molla, Claudia Joffily Parahyba, Wanessa Miranda-Silva, Paula F. Asprino, Luciana Tucunduva, Vanderson Rocha, Yana Novis, Celso Arrais-Rodrigues, Anamaria A. Camargo, Eduardo R. Fregnani

**Affiliations:** 1 Centro de Oncologia Molecular, Hospital Sírio Libanês, Rua Prof. Daher Cutait, 69, São Paulo, SP Brazil; 2 Departamento de Bioquímica, Instituto de Química, Universidade de São Paulo, São Paulo, SP Brazil; 3 Hospital Nove de Julho, São Paulo, SP Brazil; 4 Centro de Oncologia, Hospital Sírio Libanês, São Paulo, SP Brazil; 5 Hospital das Clínicas da Faculdade de Medicina, Universidade de São Paulo/ICESP, São Paulo, SP Brazil; 6 Churchill Hospital, NHS-BT, Oxford, UK

**Keywords:** Oral diseases, Oral microbiology

## Abstract

Oral mucositis (OM) is a complex acute cytotoxicity of antineoplastic treatment that affects 40–85% of patients undergoing hematopoietic stem-cell transplantation. OM is associated with prolonged hospitalization, increased extensive pharmacotherapy, need for parenteral nutrition, and elevated treatment costs. As OM onset relates to the mucosal microenvironment status, with a particular role for microbiota-driven inflammation, we aimed to investigate whether the oral mucosa microbiota was associated with the clinical course of OM in patients undergoing allogeneic hematopoietic stem-cell transplantation. We collected oral mucosa samples from 30 patients and analyzed the oral mucosa microbiota by 16S rRNA sequencing. A total of 13 patients (43%) developed ulcerative OM. We observed that specific taxa were associated with oral mucositis grade and time to oral mucositis healing. *Porphyromonas* relative abundance at preconditioning was positively correlated with ulcerative OM grade (Spearman ρ = 0.61, P = 0.028) and higher *Lactobacillus* relative abundance at ulcerative OM onset was associated with shortened ulcerative OM duration (P = 0.032). Additionally, we generated a machine-learning-based bacterial signature that uses pre-treatment microbial profiles to predict whether a patient will develop OM during treatment. Our findings suggest that further research should focus on host-microbiome interactions to better prevent and treat OM.

## Introduction

Allogeneic hematopoietic stem-cell transplantation (allo-HSCT) recipients undergo high doses of chemotherapy and, sometimes, total body irradiation during the conditioning regimen. During this period, they frequently experience treatment toxicities and immunity imbalance, affecting their quality of life^1^. Oral mucositis (OM) is a clinically relevant toxicity in the allo-HSCT setting, with incidences ranging from 15% (reduced intensity conditioning regimen) to 60–100% (myeloablative regimen)^2,3^. The reasons why OM is detrimental are manifold. It can cause treatment delay, early discontinuation of chemotherapy, prolonged hospitalization, extended use of analgesics, and even life-threatening complications^1,2^.

Clinically, severe OM presents as an ulcer with reddish borders covered by a white pseudomembrane colonized by bacteria. OM onset in allo-HSCT recipients occurs 5–7 days after the start of the conditioning regimen^4^. Established therapies for OM involve promoting epithelial healing and reducing microbial load. Examples include basic oral care, anti-inflammatory agents, photobiomodulation, cryotherapy, and antimicrobial agents^1^.

Although not yet fully elucidated, the pathophysiology of OM is multifactorial. It involves injuries to the epithelial and submucosal tissues through complex pro-inflammatory cascades. Besides, different factors can act directly on cell homeostasis affecting apoptosis and cell renewal, resulting in cell atrophy and ulceration^4^.

Contributing to this complexity, there are many risk factors for OM. Genetic variables (e.g., immunogenetic variants), demographic data, tumor-related variables (e.g., malignant potential), and treatment history, among other factors, can affect the patient’s risk of developing OM during allo-HSCT^5^. Although most risk factors associated with the incidence of OM cannot be changed, there are factors in the oral microenvironment that could be modulated—such as the oral microbiota^6–8^.

In this study, we evaluated how the oral mucosal microbiota changes, from preconditioning to the OM healing, in addition to describing the changes in diversity and composition along the allo-HSCT, we also analyzed the microbiota of patients who had not developed OM. We found specific oral commensal bacterial genera associated with OM grade and duration, and generated a machine-learning-based bacterial signature to predict whether a patient will develop oral mucositis during treatment. Identifying modifiable OM risk factors can aid personalized oral care for OM prevention and treatment.

## Results

### Patient characteristics and OM clinical course

**Figure 1:**
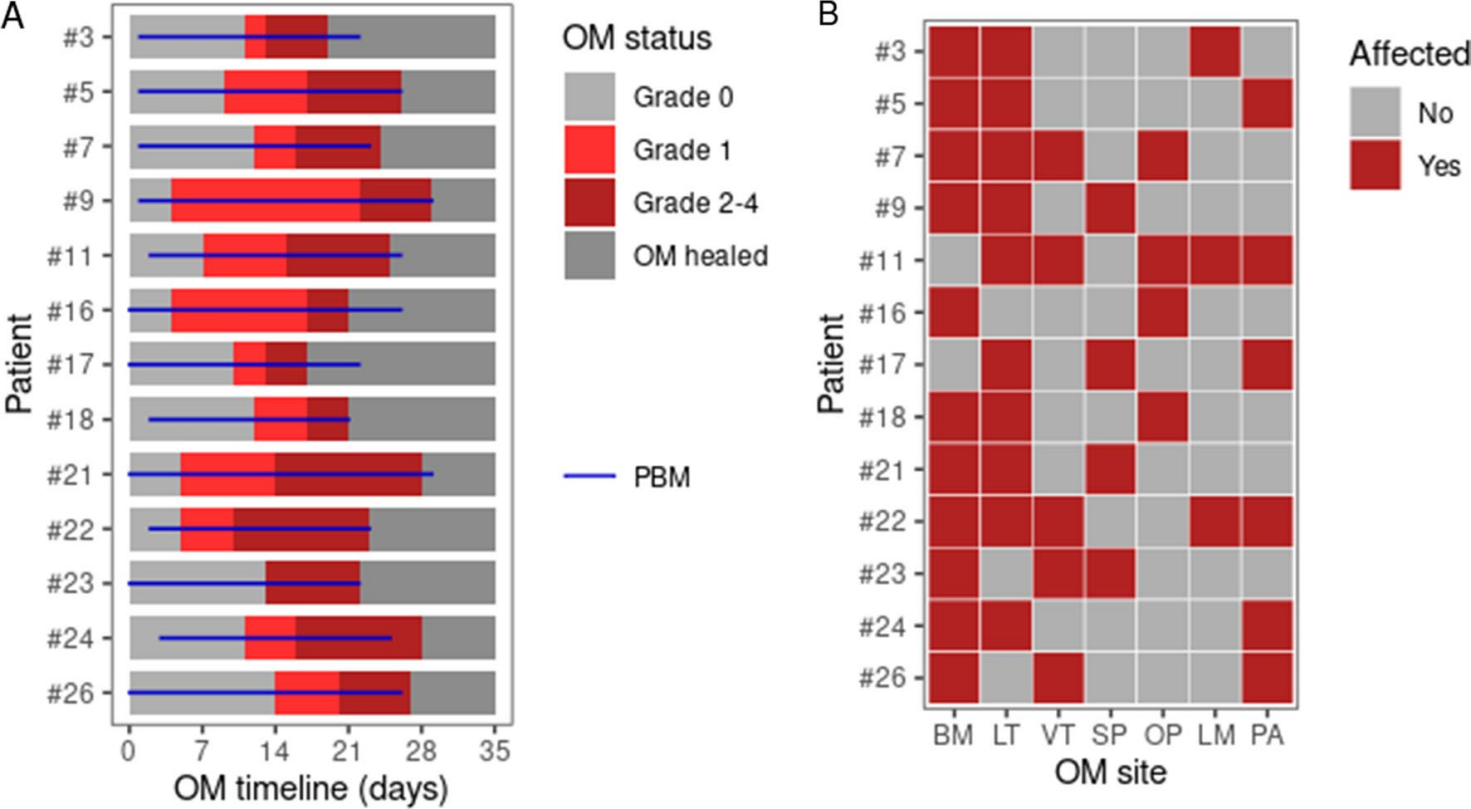
Oral mucositis (OM) timeline and sites affected by OM for each patient. (**A**) OM timeline in days for each OM patient. The OM grade along the timeline is indicated by a color scheme and the use of photobiomodulation (PBM) is indicated by a blue horizontal line. (**B**) Heatmap with the oral sites affected by OM for each patient. *BM* buccal mucosa, *LT* lateral tongue, *VT* ventral tongue, *SP* soft palate. *OP* oropharynx, *LM* labial mucosa, *PA* palatoglossal arches.

A total of 30 patients undergoing allo-HSCT in our institution between January 2016 and April 2018 were enrolled in this study (Table S1). Patients with periodontal disease were not included. Eighteen patients developed OM during the conditioning regimen, out of which 5 displayed only non-ulcerative OM (OM grade = 0 and 1) and 13 eventually displayed ulcerative OM (OM grade ≥ 2) during follow-up. Most patients (29/30) used broad-spectrum antibiotics during the conditioning regimen, so that there was no clear association between OM incidence and broad-spectrum antibiotic use before OM onset. The timeline of OM status for these patients, as well as the period of photobiomodulation treatment, is provided in Fig. 1A. The median number of photobiomodulation sessions were 25 (one session per day). The number of affected sites per patient varied between 2 and 5 (Fig. 1B), with buccal mucosa representing the most affected site (11/13 patients). Patients who developed ulcerative OM showed a non-significant trend (P = 0.064) towards showing non-ulcerative OM symptoms earlier during follow-up (Fig. S1). Most ulcerative OM patients (12/13) used broad-spectrum antibiotics during ulcerative OM. Due to the focus of this study on ulcerative OM, we will refer to it hereinafter simply as OM.

### Characterization of the oral microbiota during OM

We evaluated the oral microbiota of the 13 OM patients during the OM clinical course. For each patient, 16S amplicon sequencing of oral samples was performed at preconditioning (P), oral mucositis onset (MO), and when oral mucositis was healed (MH). One sample did not achieve a satisfactory number of reads and was discarded (patient #5, MH).

**Figure 2:**
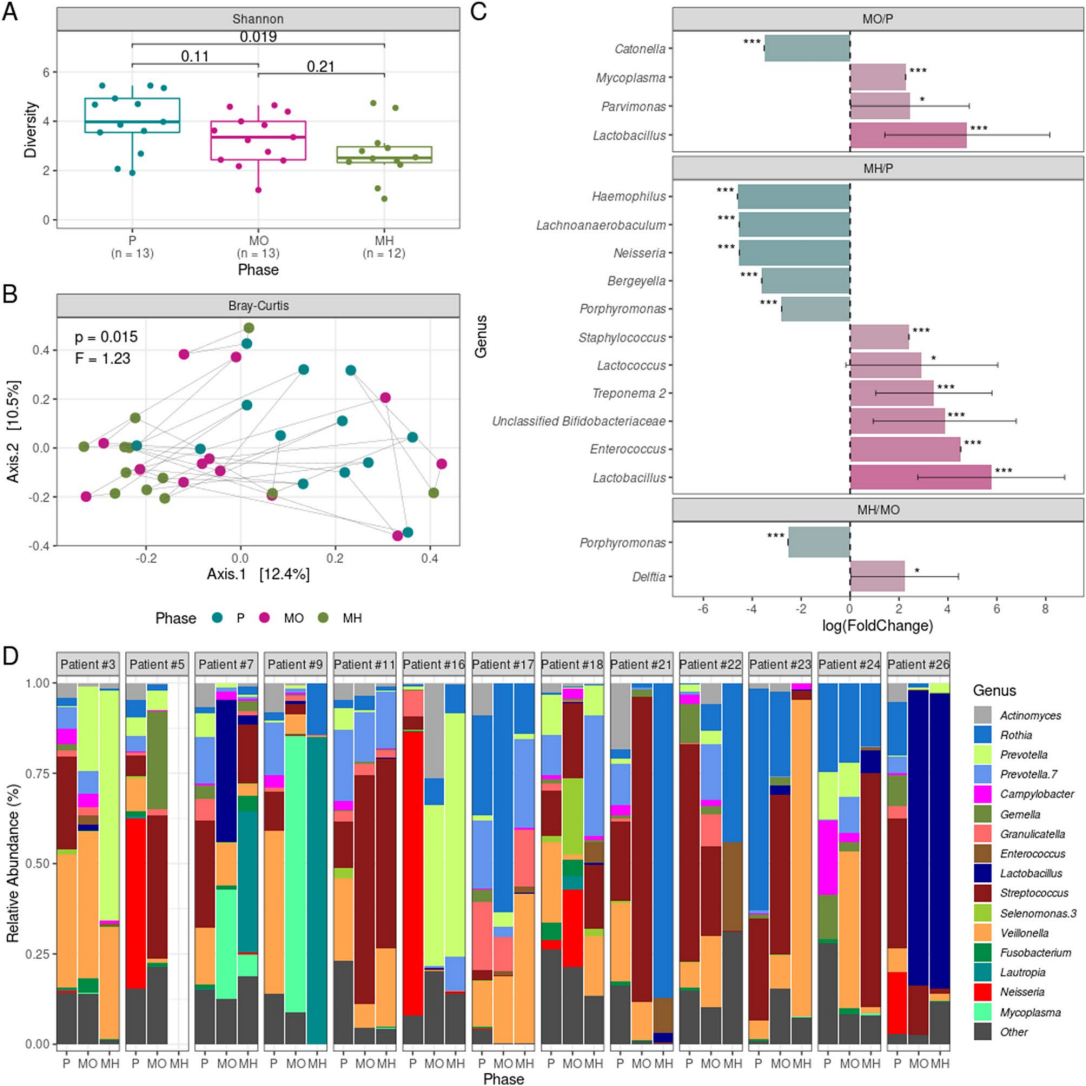
Changes in diversity and composition during oral mucositis (OM) clinical course. (**A**) Alpha-diversity boxplots at preconditioning (P), OM onset (MO), and OM healed (MH). Shannon was used as alpha-diversity metric. Statistical significance was evaluated by the Mann–Whitney U test, with P-values indicated. The boxes highlight the median value and cover the 25th and 75th percentiles, with whiskers extending to the more extreme value within 1.5 times the length of the box. (**B**) Principal coordinates analysis showing changes in composition during OM clinical course (beta-diversity). Bray–Curtis was used as beta-diversity metric. Samples from the same patient are linked by a gray line. Statistical significance was evaluated by the PERMANOVA test, with P- and F-values indicated. (**C**) Significant alterations in genera abundances between collection timepoints according to the ANCOM-BC test. *Adjusted P-value < 0.05; ***adjusted P-value < 0.001. (**D**) Genera relative abundances for each OM patient across collection timepoints. Only genera with > 1% relative abundance in > 25% of the samples or > 20% relative abundance in at least one sample are shown.

Alpha-diversity significantly differed only between P and MH, although we observed a non-significant alpha-diversity decrease from P to MO and a further decrease from MO to MH (Fig. 2A). Moreover, beta-diversity significantly differed between timepoints, indicating that the oral microbiota possesses different bacterial compositions during OM clinical course (Fig. 2B).

To investigate which taxa were driving those differences in composition, we performed a differential abundance analysis at genus level with ANCOM-BC (Fig. 2C). The overall taxonomic composition at genus level for each patient during OM clinical course is provided in Fig. 2D.

We identified several differentially abundant genera between timepoints. For instance, *Lactobacillus* is on average 120× more abundant in MO compared to P samples (Fig. 2C). This is also clear in terms of relative abundance, where patients #3, #7, and #26 show increased *Lactobacillus* relative abundance to the detriment of other genera in MO samples compared to P samples (Fig. 2D). A decrease in *Catonella* and increases in *Mycoplasma* and *Parvimonas* also marked the progression from P to MO (Fig. 2C).

Most of the differences were observed in the P vs. MH comparison, including increases in *Lactobacillus* and *Enterococcus* and decreases in *Haemophilus* and *Lachnoaerobaculum* (Fig. 2C). When comparing MO and MH samples, there were only two significantly differentially abundant genera between timepoints (Fig. 2C). While *Delftia* increased in abundance from MO to MH, *Porphyromonas* decreased. *Porphyromonas* is also more abundant at P in comparison to MH. In fact, in both comparisons, *Porphyromonas* was classified by ANCOM-BC as a structural zero, meaning it is not only more abundant in MO or P in comparison to MH, but that it is totally absent in MH samples.

### Preconditioning oral microbiota and risk of OM development

Next, we evaluated whether the P oral microbiota was informative on the risk of OM development. To do so, we profiled the microbiota of P oral samples from all patients, which included 17 samples from patients that did not develop OM (OM-free) and 13 patients with OM. One sample from a patient of the OM-free group did not achieve a satisfactory number of reads and was discarded.

**Figure 3:**
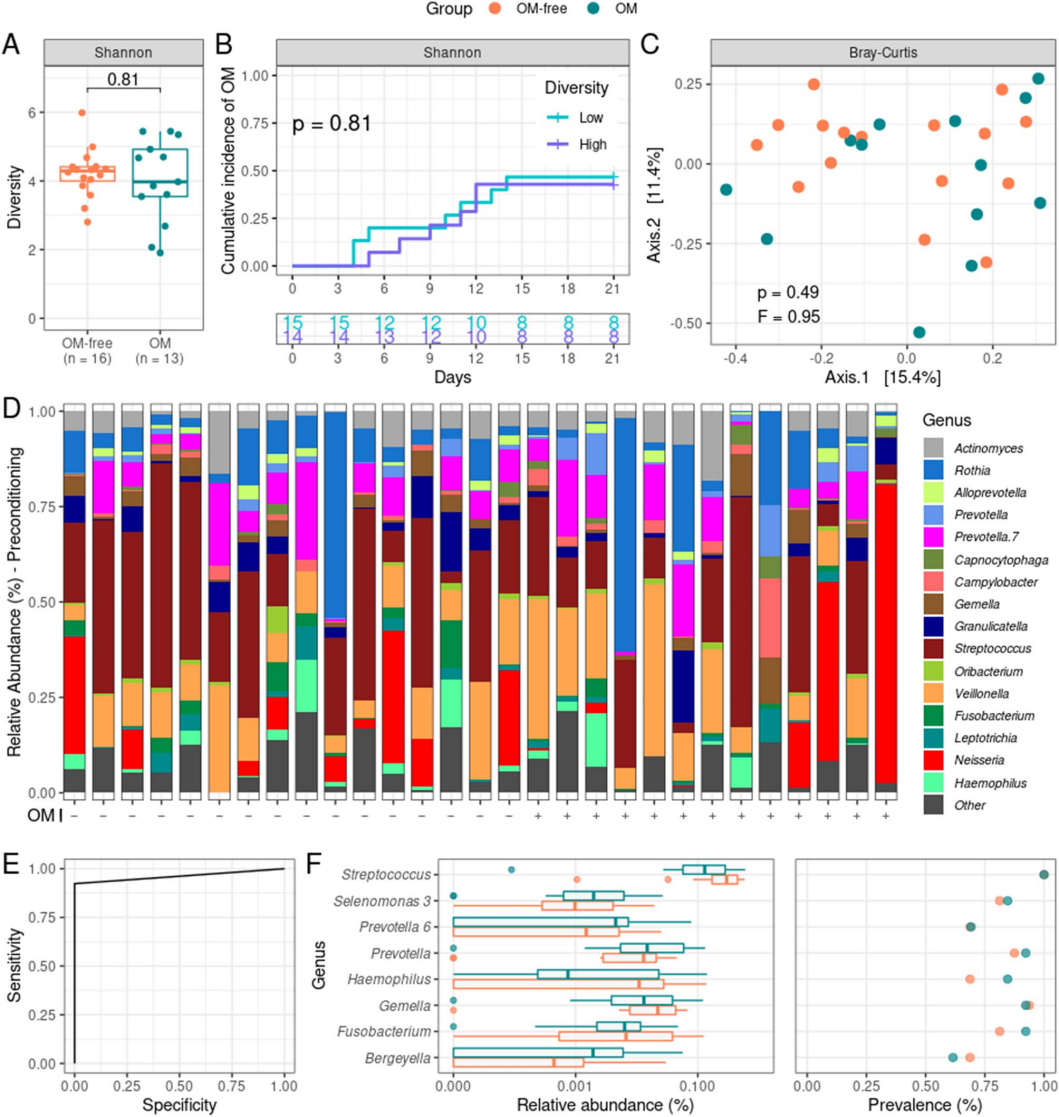
Comparisons between oral mucositis (OM) patients and OM-free patients at preconditioning (P). (**A**) Alpha-diversity boxplots at P for OM and OM-free patients. Shannon was used as alpha-diversity metric. Statistical significance was evaluated by the Mann–Whitney U test, with P-value indicated. The boxes highlight the median value and cover the 25th and 75th percentiles, with whiskers extending to the more extreme value within 1.5 times the length of the box. (**B**) Cumulative incidence curves of OM with patients stratified by alpha-diversity level (low/high, based on median Shannon index) at preconditioning. The number of patients at risk is shown. Statistical significance was evaluated by the log-rank test, with P-value indicated. (**C**) Principal coordinates analysis comparing compositions at P of OM and OM-free patients (beta-diversity). Bray–Curtis was used as a beta-diversity metric. Statistical significance was evaluated by the PERMANOVA test, with P- and F-values indicated. (**D**) Genera relative abundances at P for OM and OM-free patients. Patients are sorted based on OM categories (OM-free/OM: −/+), as indicated by x-axis labels. Only genera with > 1% relative abundance in > 25% of the samples or > 20% relative abundance in at least one sample are shown. (**E**) Receiver-operating characteristic curve for a support vector machine model (SVM) for classifying patients into OM and OM-free categories based on P oral microbiota data. The model was built based on the relative abundances of eight genera at P. (**F**) Relative abundances boxplots (left) and prevalence (right) for OM and OM-free patients of the eight genera at P used in the SVM model. A symlog scale was used in the x-axis of the relative abundances plot, with 10^–5^ as linearity threshold.

There was no difference in alpha-diversity between OM-free and OM patients at P (Fig. 3A). Accordingly, low and high alpha-diversity patients (stratified based on median Shannon index) showed no difference in OM cumulative incidence (Fig. 3B). Furthermore, oral microbiota compositions at P between OM-free and OM patients did not differ, as evaluated by a beta-diversity analysis (Fig. 3C). In line with this result, there were no significant differences in genera abundances between groups (Table S2). This can be visualized by relative abundance plots with patients sorted by OM incidence, where no signal of genera associated with OM-free or OM patients is apparent (Fig. 3D). We further confirmed that none of the P genera was associated with the OM risk using Cox regression analysis (Table S3).

To evaluate whether a signature of P genera was associated with the OM risk, we built a SVM model based on all P samples. A 96.6% accuracy (sensitivity: 92.3%; specificity: 100%) in predicting OM onset was achieved when evaluating a signature of eight genera (Fig. 3E). Differences in relative abundance and prevalence between groups for these eight genera are detailed in Fig. 3F. We also evaluated this model by leave-one-out cross-validation, showing good generalizability (82.8% mean accuracy).

### Genera associated with OM clinical course

**Figure 4:**
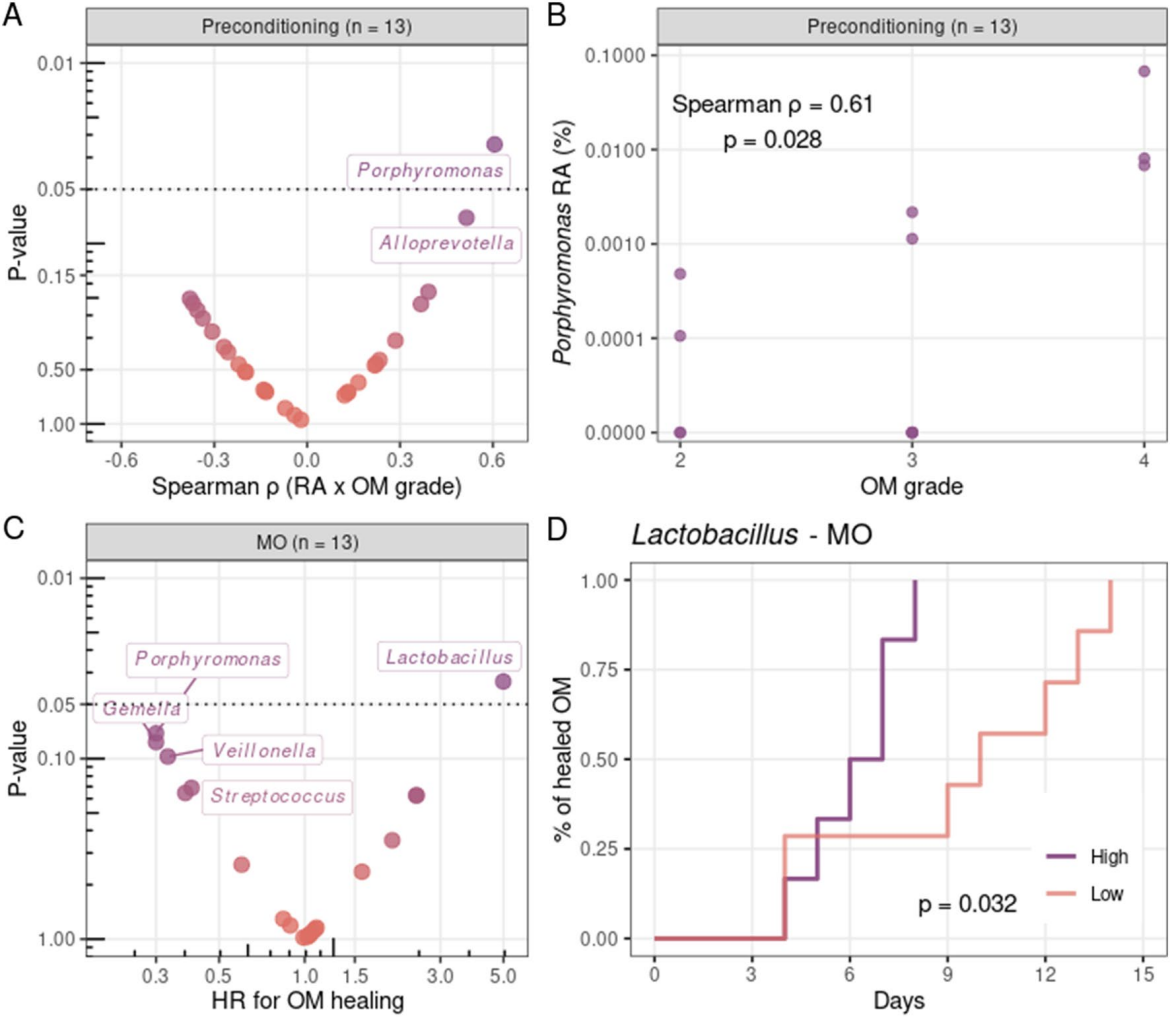
Genera associated with oral mucositis (OM) grade and OM healing. (**A**) Volcano plot (Spearman ρ vs. P-value) depicting correlations between the highest OM grade and genera relative abundances at preconditioning (P). (**B**) Spearman correlation between the highest OM grade and *Porphyromonas* relative abundance at P. Spearman ρ and P-value are indicated. *RA* relative abundance. (**C**) Volcano plot (Cox hazard ratio vs. P-value) of the risk analysis for the association of OM healing with genera relative abundance at OM onset (MO). (**D**) Cumulative incidence curves of healed OM with patients stratified by *Lactobacillus* relative abundance (low/high, based on median value) at MO. Statistical significance was evaluated by the log-rank test, with P-value indicated. In (**A,C**), only genera present (non-zero relative abundance) in > 50% of the samples were evaluated. Only genera with P-value < 0.15 are indicated explicitly.

Finally, we investigated whether there were oral genera associated with OM grade and time to OM healing. When considering all patients (including OM grade < 2), even though *Streptococcus* relative abundance at P marginally correlated with lower grade OM (P = 0.06), none of the genus at P significantly correlated with OM grade during follow-up (Table S4). However, when considering only patients with OM grade ≥ 2, we observed that *Porphyromonas* relative abundance at P was significantly correlated with OM grade (Table S5, Fig. 4A). In fact, the top-three patients in terms of *Porphyromonas* relative abundance at P were the only patients that developed OM grade = 4 (Fig. 4B).

Next, we evaluated whether genera relative abundances at MO were associated with the time to OM healing using Cox regression analysis, with MO as the baseline. We found that *Lactobacillus* relative abundance at MO was significantly associated with time to OM healing (Table S6, Fig. 4C), with patients classified (based on median value) as having high *Lactobacillus* relative abundance at MO showing earlier OM healing (median time: 6 vs. 10 days; Fig. 4D).

## Discussion

Initially, OM was considered a result of non-specific cell death. Currently, a series of biological events explains the progression of ulceration^9^. OM development can be divided into two stages. The initiation stage consists of chemoradiotherapy-induced DNA damage, prompting the generation of reactive oxygen species by basal epithelial cells. Consequently, inflammation-associated pathways are triggered. The most studied pathway in the pathophysiology of OM is the NF-κB signaling pathway, responsible for the expression of molecules that modulate stress, cell adhesion, apoptosis, and inflammation. Chemotherapy and radiotherapy also have indirect effects on the oral mucosa through activation of the ceramide pathway, leading to fibrolysis and production of metalloproteinases. In the second stage, named signal amplification phase, some pathways activated in the initiation stage promote higher levels of inflammation in the damaged epithelial tissue^9,10^.

However, despite the huge impact of OM on the quality of life of cancer patients, it is still not clear how a patient’s personal characteristics/markers can influence the incidence of OM^5^. In this work, we describe how the bacterial composition of the oral mucosa could be used as a predictive biomarker for OM in patients undergoing allo-HSCT. Oral commensals such as *Porphyromonas* and *Lactobacillus* are associated with the OM severity and healing period. Additionally, we provide a characterization of the oral mucosa microbiota dynamics during allo-HSCT with a detailed data collection of OM duration, grade, and anatomical sites affected.

There are no preventive strategies based on a patient’s microenvironmental characteristics. OM preventive strategies are based on oral hydration to decrease mucosal friability, photobiomodulation to increase mucosal repair potential, and oral hygiene for unspecific microbial control. In this context, omics-based analyses can help elucidate the influence of the oral microbiota on OM onset and provide evidence to support future studies on microbial modulation as a preventive and curative strategy. Beyond oral side effects, our previous study showed an association between low bacterial diversity of oral mucosa microbiota at preconditioning and a higher risk of relapse^11^.

*Porphyromonas* is known to be a key-pathogen of chronic periodontal disease, being found in 85% of periodontal pockets^12^. Additionally, its impact on systemic diseases has gained increased attention in the literature, including associations with inflammatory bowel disease^13^ and Alzheimer’s disease^14^. *Porphyromonas gingivalis* can manipulate the host’s innate immune response, being able to adapt, invade and survive. Beyond the activation of inflammatory pathways, *Porphyromonas gingivalis* pathogenicity can be explained by its survival strategy that circumvents the immune system by invading host cells. Invasion occurs mainly through the interaction between the fimbriae and B1 integrins of host cells, which triggers cytoskeletal restructuring, allowing bacterial internalization. Noteworthy, invasion does not trigger cell apoptosis, allowing bacterial survival and replication within the host cell^12,15,16^. Our results showing that the relative abundance of *Porphyromonas* at preconditioning is correlated with the highest OM grade presented during follow-up reinforce the importance of studying this genus in the context of oral care in hospitalized cancer patients. Furthermore, we found that *Porphyromonas* is virtually absent in MH samples. Although causality cannot be evaluated, this result suggests *Porphyromonas* clearance may be necessary for OM healing, an intriguing hypothesis that also demands further investigation.

The use of probiotics containing *Lactobacillus* is being evaluated to prevent OM severity in head and neck cancer patients^6–8^. One phase II study prescribing *Lactobacillus brevis CD2* for HSCT recipients reported lower grades of OM. The putative mechanism of action involves the production of arginine deiminase by *Lactobacillus brevis CD2,* which downregulates the pro-inflammatory nitric oxide pathway^17^. Our results showing that the relative abundance of wild/natural *Lactobacillus* is associated with a faster ulcerative OM healing time supports future clinical trials in patients undergoing allo-HSCT.

Other studies analyzed the role of the oral microbiota in OM during oncohematologic treatment^18–22^. Onesuch study showed a decrease in bacterial diversity during transplantation and a greater abundance of specific genera only in patients who used methotrexate prior to allo-HSCT^19^. In another study, a decrease in diversity was noted in patients without ulcerative oral mucositis^18^. One work focused on patients undergoing allo-HSCT and OM severity, even though by evaluating saliva samples. They found associations between the relative abundance of *Kingella* and *Atopobium* in saliva and OM severity^19^. In our study, these genera were not associated with OM parameters, possibly due to the evaluation herein of oral mucosa samples rather than saliva. A long-term analysis of saliva microbiome in allo-HSCT showed reestablishment of bacterial diversity months after stem-cell infusion. And patients who developed OM had lower diversity in the third week when compared with patients without OM^23^.

Besides describing variations in the oral microbiota during OM clinical course, we also evaluated whether oral microbiota composition could be used as a biomarker for OM incidence. Among other results, we provide for the first time a machine-learning-based bacterial signature for predicting OM. This signature includes only eight genera: *Streptococcus*, *Selenomonas 3*, *Prevotella 6*, *Prevotella*, *Haemophilus*, *Gemella*, *Fusobacterium*, and *Bergeyella*—possible research targets for OM onset. Validation cohorts are needed to confirm the clinical value of this bacterial signature. Further studies will also be needed to overcome the limitations of our study, such as the lack of longitudinally collected samples from OM-free patients and small sample size.

Oral care is an essential part of the oncologic treatment, as it maintains patient’s quality of life, decreases the use of analgesics and shortens hospitalization period. Predictive analysis is a fundamental part of precision medicine and supports the innovation of clinical guidelines. Our study highlights the role of commensal oral bacteria in OM clinical course. It also demonstrates the importance of characterizing the oral microbiota in oncologic patients for improving clinical care. Further, more powered studies will be necessary to evaluate the influence of commensals and pathogens in the pathophysiology of OM.

## Materials and methods

### Sample collection

Enrolled patients underwent allo-HSCT at Hospital Sírio-Libanês (São Paulo/Brazil) between 2016 and 2018. The study was approved by the local ethics committee (Comite de Ética em Pesquisa—Hospital Sírio-Libanês (#HSL 2016-08)), according to the Declaration of Helsinki, and all patients provided informed consent before sample collection. No tissue was procured from prisoners in this study.

The oral mucosa sample was collected with a sterile swab on bilateral buccal mucosa, alveolar mucosa of the jaws, and tongue dorsum. Samples were collected at preconditioning (before conditioning regimen), ulcerative OM onset, and when OM ulcerations were healed (no sign of ulceration). Patients did not perform oral hygiene for at least 6 h before sample collection.

### Institutional standard antimicrobial prophylaxis

The standard antimicrobial prophylaxis in our institution included oral levofloxacin and/or sulfamethoxazole-trimethoprim, acyclovir, and antifungal prophylaxis according to the patient’s risk of fungal infection (low risk: fluconazole; high risk: voriconazole).

### Oral care and photobiomodulation

All patients were examined and treated by two trained professionals of the oral medicine department of our institution following the MASCC/ISOO Guideline for Cancer Patients^24^. The standard oral hygiene protocol was fluoride toothpaste and 0.12% chlorhexidine (CHX) mouthwash. The topical CHX was administered once a day. The photobiomodulation protocol was performed with low-level laser equipment (Laser XT Therapy, DMC, São Carlos, Brazil) at a wavelength of 660 nm (spot size = 0.028 cm^2^; 100mW of power) irradiating 64 points of the oral mucosa, covering buccal mucosa, mucobuccal fold, palatoglossal arches, soft palate, labial mucosa, tongue (lateral and ventral). The irradiation ranged between 1 and 2 J/point for preventive and curative treatment for oral lesions, respectively.

### DNA extraction and 16S rRNA amplicon sequencing

Bacterial cells were recovered from oral mucosa swabs using TE buffer and 6 μL PureLink RNAse A (20 mg/mL, Thermo Fisher Scientific, Waltham, MA, USA). DNA was extracted using QIAamp DNA Blood Mini Kit (Qiagen, Hilden, Germany) according to the manufacturer’s protocol (DNA Purification from Blood or Body Fluids) and stored at − 80 °C. Pre-validated primers and 12.5 ng DNA were used to amplify the 16S rRNA hypervariable regions V3–V4^25^. Amplicons were sequenced as described elsewhere^26^ on an Illumina MiSeq platform (Illumina, San Diego, CA, USA).

### Bioinformatics pipeline

Reads were processed with QIIME 2^27^ following the DADA2 pipeline^28^ to generate Amplicon Sequencing Variants (ASVs). Chimeric ASVs were filtered out with VSEARCH^29^ by using the SILVA database as reference^30^. The taxonomic assignment of ASVs was performed with VSEARCH and SILVA. ASVs not assigned to bacteria were removed. After read filtering steps, samples with < 1000 reads were discarded. Next, microbiota analysis was performed using custom R scripts^31^.

### Microbiota analyses

Libraries were normalized to 6256 reads by Scaling with Ranked Subsampling^32^ with the R package *SRS*^33^ to account for variable sequencing depth prior to diversity analysis. Alpha-diversity was calculated at ASV level with the QIIME 2 plugin *q2-diversity* using the Shannon index^34^. Differences in alpha-diversity between groups were evaluated using the Mann–Whitney U test. Beta-diversity was calculated at ASV level with the R package *phyloseq*^35^ using Bray–Curtis dissimilarity index^36^. Compositional differences between groups were represented by Principal Coordinate Analysis and evaluated using the PERMANOVA test^37^.

In genera relative abundance plots (generated with the R package *ggplot2*^38^) only genera with > 1% relative abundance in > 25% of the samples or > 20% relative abundance in at least one sample are shown. Differential abundance of genera between groups was evaluated with ANCOM-BC^39^. Genera with log (FoldChange) > 2 between groups and P < 0.05 after Bonferroni correction were considered statistically significant.

Only genera present (non-zero relative abundance) in > 50% of the samples were evaluated in the associations between genera relative abundance and OM clinical course. Associations between genera relative abundance and OM stage were evaluated using Spearman correlation. Associations between genera relative abundance and time to OM development (with the starting day of the conditioning regimen as reference) or time to OM healing were evaluated by stratifying patients into low and high groups (based on median genus relative abundance) and estimating the Cox proportional hazards between groups with the R package survival^40^. The same approach was used to associate alpha-diversity with time to OM development, with patients stratified into low and high alpha-diversity groups based on the median Shannon index. Kaplan–Meier curves were generated with the R package *survminer*^41^.

The support vector machine (SVM) model was generated with the R package *kernlab*^42^. All preconditioning samples were included and only genera present in > 50% of preconditioning samples were considered. The model was tested using the leave-one-out cross-validation approach. The final model was built with the number of genera (n = 8) and the cost (C = 10) that maximized cross-validation accuracy.

## Supplementary Information

Supplementary Information.

## Data Availability

Sequencing data were deposited in the European Nucleotide Archive (ENA) at EMBL-EBI under Accession Number PRJEB49175.
