# Supplementary material for: Commensal oral microbiota impacts ulcerative oral mucositis clinical course in allogeneic stem cell transplant recipients
Source: Sci Rep. 2022 Oct 20;12:17527. doi: 10.1038/s41598-022-21775-3 (PMC9584897; doi:10.1038/s41598-022-21775-3)
Supplement: Supplementary file 1 — Supplementary Information. [file 41598_2022_21775_MOESM1_ESM.pdf]

SUPPLEMENTAL MATERIAL

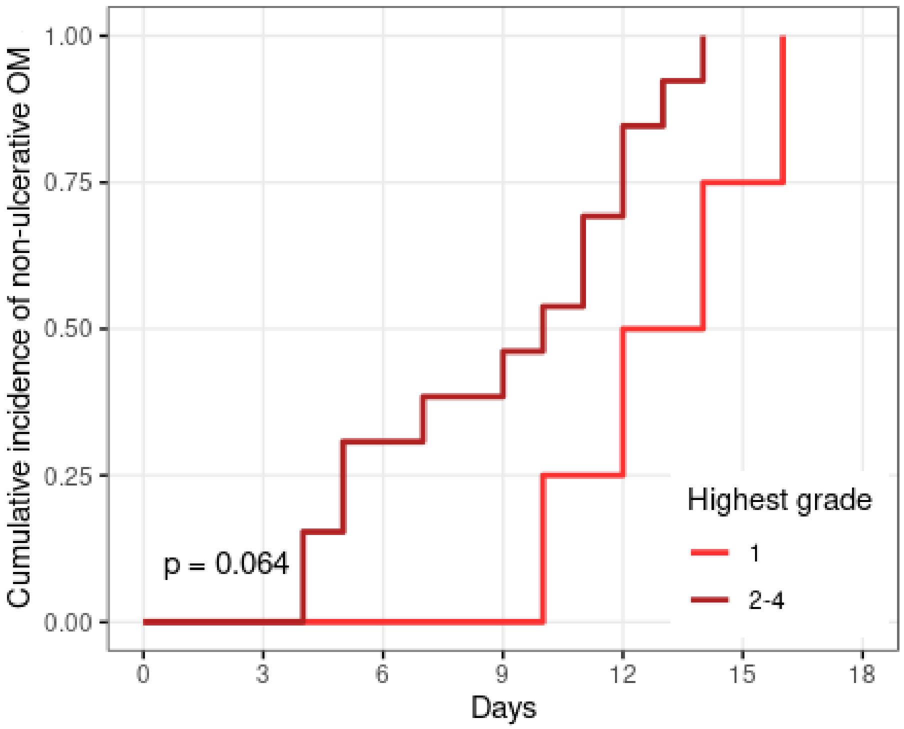

Figure S1: Cumulative incidence curves of non-ulcerative OM with patients stratified by the highest OM grade during OM clinical course (1/2-4). Statistical significance was evaluated by the log-rank test, with P-value indicated.

	n = 30
**Age in years (median, range)**	50 (19-73)
**Sex (male)**	16 (53%)
**Oral Mucosits Grades (WHO)**	
Grade 0	12 (40%)
Grade I	5 (17%)
Grade II	4 (13%)
Grade III	6 (20%)
Grade IV	3 (10%)
**Conditioning Intensity**	
Reduced Intensity	18 (60%)
**Total Body Irradiation**	11 (37%)
**Alkylating Agents**	
Busulfan	15 (50%)
Cyclophosphamide	10 (33%)
Melphalan	4 (13%)
Bendamustine	1 (3%)
Treosulfan	1 (3%)
**Graft Source**	
Bone Marrow	10 (33%)
Peripheal Blood	20 (67%)
**Underlying Disease**	
Acute Myeloid Leukemia	18 (60%)
Acute Lymbhoblastic Leukemia	7 (23%)
Non-hodgkin Lymphoma	5 (17%)
Myelodysplastic Syndrome	4 (13%)
Chronic Lymphocytic Leukemia	1 (3%)
Chronic Myeloid Leukemia	2 (3%)
Multiple Myeloma	3 (3%)
**Graft-versus-Host Disease Prophylaxis**	
Mycophenolate+Cyclosporin A	11 (37%)
Methotrexate+Cysclosporin A	10 (33%)
Mycophenolate+Cyclosporin A+Cyclophosphamide	9 (30%)

Table S1: Clinical characteristics of study patients.

Genus	log(FoldChange)	P-value	Adjusted P-value	Differentially abundant
D_0__Bacteria;D_1__Actinobacteria;D_2__Actinobacteria;D_3__Actinomycetales;D_4__Actinomycetaceae;D_5__Actinomyces	-0,59	0,46	1	No
D_0__Bacteria;D_1__Actinobacteria;D_2__Actinobacteria;D_3__Actinomycetales;D_4__Actinomycetaceae;D_5__F0332	0,79	0,00	0	No
D_0__Bacteria;D_1__Actinobacteria;D_2__Actinobacteria;D_3__Bifidobacteriales;D_4__Bifidobacteriaceae;D_5__Bifidobacterium	-0,85	0,00	0	No
D_0__Bacteria;D_1__Actinobacteria;D_2__Actinobacteria;D_3__Bifidobacteriales;D_4__Bifidobacteriaceae;__	0,59	0,00	0	No
D_0__Bacteria;D_1__Actinobacteria;D_2__Actinobacteria;D_3__Micrococcales;D_4__Micrococcaceae;D_5__Rothia	-0,24	0,76	1	No
D_0__Bacteria;D_1__Actinobacteria;D_2__Coriobacteriia;D_3__Coriobacteriales;D_4__Atopobiaceae;D_5__Atopobium	-0,29	0,74	1	No
D_0__Bacteria;D_1__Bacteroidetes;D_2__Bacteroidia;D_3__Bacteroidales;D_4__Porphyromonadaceae;D_5__Porphyromonas	-0,70	0,54	1	No
D_0__Bacteria;D_1__Bacteroidetes;D_2__Bacteroidia;D_3__Bacteroidales;D_4__Prevotellaceae;D_5__Alloprevotella	-0,07	0,95	1	No
D_0__Bacteria;D_1__Bacteroidetes;D_2__Bacteroidia;D_3__Bacteroidales;D_4__Prevotellaceae;D_5__Prevotella	0,85	0,28	1	No
D_0__Bacteria;D_1__Bacteroidetes;D_2__Bacteroidia;D_3__Bacteroidales;D_4__Prevotellaceae;D_5__Prevotella 2	-0,33	0,72	1	No
D_0__Bacteria;D_1__Bacteroidetes;D_2__Bacteroidia;D_3__Bacteroidales;D_4__Prevotellaceae;D_5__Prevotella 6	0,51	0,62	1	No
D_0__Bacteria;D_1__Bacteroidetes;D_2__Bacteroidia;D_3__Bacteroidales;D_4__Prevotellaceae;D_5__Prevotella 7	-0,02	0,98	1	No
D_0__Bacteria;D_1__Bacteroidetes;D_2__Bacteroidia;D_3__Bacteroidales;D_4__Tannerellaceae;D_5__Tannerella	1,19	0,00	0	No
D_0__Bacteria;D_1__Bacteroidetes;D_2__Bacteroidia;D_3__Flavobacteriales;D_4__Flavobacteriaceae;D_5__Capnocytophaga	1,00	0,33	1	No
D_0__Bacteria;D_1__Bacteroidetes;D_2__Bacteroidia;D_3__Flavobacteriales;D_4__Weeksellaceae;D_5__Bergeyella	0,56	0,60	1	No
D_0__Bacteria;D_1__Epsilonbacteraeota;D_2__Campylobacteria;D_3__Campylobacterales;D_4__Campylobacteraceae;D_5__Campylobacter	0,38	0,60	1	No
D_0__Bacteria;D_1__Firmicutes;D_2__Bacilli;D_3__Bacillales;D_4__Family XI;D_5__Gemella	-0,31	0,75	1	No
D_0__Bacteria;D_1__Firmicutes;D_2__Bacilli;D_3__Lactobacillales;D_4__Carnobacteriaceae;D_5__Granulicatella	-0,95	0,37	1	No
D_0__Bacteria;D_1__Firmicutes;D_2__Bacilli;D_3__Lactobacillales;D_4__Lactobacillaceae;D_5__Lactobacillus	-0,17	0,85	1	No
D_0__Bacteria;D_1__Firmicutes;D_2__Bacilli;D_3__Lactobacillales;D_4__Streptococcaceae;D_5__Lactococcus	1,38	0,00	0	No
D_0__Bacteria;D_1__Firmicutes;D_2__Bacilli;D_3__Lactobacillales;D_4__Streptococcaceae;D_5__Streptococcus	-0,70	0,40	1	No
D_0__Bacteria;D_1__Firmicutes;D_2__Clostridia;D_3__Clostridiales;D_4__Family XI;D_5__Parvimonas	-0,71	0,31	1	No
D_0__Bacteria;D_1__Firmicutes;D_2__Clostridia;D_3__Clostridiales;D_4__Family XIII;D_5__Mogibacterium	0,96	0,00	0	No
D_0__Bacteria;D_1__Firmicutes;D_2__Clostridia;D_3__Clostridiales;D_4__Family XIII;D_5__[Eubacterium] nodatum group	0,16	0,83	1	No
D_0__Bacteria;D_1__Firmicutes;D_2__Clostridia;D_3__Clostridiales;D_4__Lachnospiraceae;D_5__Butyrivibrio 2	-0,21	0,77	1	No
D_0__Bacteria;D_1__Firmicutes;D_2__Clostridia;D_3__Clostridiales;D_4__Lachnospiraceae;D_5__Catonella	0,33	0,66	1	No
D_0__Bacteria;D_1__Firmicutes;D_2__Clostridia;D_3__Clostridiales;D_4__Lachnospiraceae;D_5__Lachnoanaerobaculum	1,09	0,21	1	No
D_0__Bacteria;D_1__Firmicutes;D_2__Clostridia;D_3__Clostridiales;D_4__Lachnospiraceae;D_5__Oribacterium	0,55	0,42	1	No
D_0__Bacteria;D_1__Firmicutes;D_2__Clostridia;D_3__Clostridiales;D_4__Lachnospiraceae;D_5__Stomatobaculum	0,46	0,57	1	No
D_0__Bacteria;D_1__Firmicutes;D_2__Clostridia;D_3__Clostridiales;D_4__Lachnospiraceae;__	0,06	0,95	1	No
D_0__Bacteria;D_1__Firmicutes;D_2__Clostridia;D_3__Clostridiales;D_4__Peptostreptococcaceae;D_5__Peptostreptococcus	0,14	0,81	1	No
D_0__Bacteria;D_1__Firmicutes;D_2__Clostridia;D_3__Clostridiales;D_4__Ruminococcaceae;D_5__Ruminococcaceae UCG-014	-1,04	0,27	1	No
D_0__Bacteria;D_1__Firmicutes;D_2__Erysipelotrichia;D_3__Erysipelotrichales;D_4__Erysipelotrichaceae;D_5__Solobacterium	0,28	0,69	1	No
D_0__Bacteria;D_1__Firmicutes;D_2__Negativicutes;D_3__Selenomonadales;D_4__Veillonellaceae;D_5__Dialister	0,67	0,00	0	No
D_0__Bacteria;D_1__Firmicutes;D_2__Negativicutes;D_3__Selenomonadales;D_4__Veillonellaceae;D_5__Megasphaera	-0,29	0,78	1	No
D_0__Bacteria;D_1__Firmicutes;D_2__Negativicutes;D_3__Selenomonadales;D_4__Veillonellaceae;D_5__Selenomonas	0,16	0,84	1	No
D_0__Bacteria;D_1__Firmicutes;D_2__Negativicutes;D_3__Selenomonadales;D_4__Veillonellaceae;D_5__Selenomonas 3	0,71	0,37	1	No
D_0__Bacteria;D_1__Firmicutes;D_2__Negativicutes;D_3__Selenomonadales;D_4__Veillonellaceae;D_5__Veillonella	-0,26	0,71	1	No
D_0__Bacteria;D_1__Fusobacteria;D_2__Fusobacteriia;D_3__Fusobacteriales;D_4__Fusobacteriaceae;D_5__Fusobacterium	0,19	0,83	1	No
D_0__Bacteria;D_1__Fusobacteria;D_2__Fusobacteriia;D_3__Fusobacteriales;D_4__Leptotrichiaceae;D_5__Leptotrichia	0,25	0,76	1	No
D_0__Bacteria;D_1__Patescibacteria;D_2__Saccharimonadia;D_3__Saccharimonadales;D_4__Saccharimonadaceae;D_5__Candidatus Saccharimonas	1,76	0,00	0	No
D_0__Bacteria;D_1__Patescibacteria;D_2__Saccharimonadia;D_3__Saccharimonadales;D_4__Saccharimonadaceae;D_5__uncultured bacterium	-0,65	0,53	1	No
D_0__Bacteria;D_1__Patescibacteria;D_2__Saccharimonadia;D_3__Saccharimonadales;D_4__uncultured bacterium;D_5__uncultured bacterium	0,89	0,00	0	No
D_0__Bacteria;D_1__Proteobacteria;D_2__Gammaproteobacteria;D_3__Betaproteobacteriales;D_4__Burkholderiaceae;D_5__Lautropia	0,60	0,42	1	No
D_0__Bacteria;D_1__Proteobacteria;D_2__Gammaproteobacteria;D_3__Betaproteobacteriales;D_4__Neisseriaceae;D_5__Kingella	1,10	0,00	0	No
D_0__Bacteria;D_1__Proteobacteria;D_2__Gammaproteobacteria;D_3__Betaproteobacteriales;D_4__Neisseriaceae;D_5__Neisseria	-0,64	0,68	1	No
D_0__Bacteria;D_1__Proteobacteria;D_2__Gammaproteobacteria;D_3__Cardiobacteriales;D_4__Cardiobacteriaceae;D_5__Cardiobacterium	-0,48	0,40	1	No
D_0__Bacteria;D_1__Proteobacteria;D_2__Gammaproteobacteria;D_3__Pasteurellales;D_4__Pasteurellaceae;D_5__Haemophilus	-0,59	0,61	1	No
D_0__Bacteria;D_1__Spirochaetes;D_2__Spirochaetia;D_3__Spirochaetales;D_4__Spirochaetaceae;D_5__Treponema 2	-0,33	0,64	1	No

Table S2: Differential abundance analysis between oral mucositis (OM) and OM-free patients at preconditioning. The analysis was performed with ANCOM-BC at genus level. No genera passed the log(FoldChange) and adjusted P-value cutoffs to be considered as significantly differentially abundant between groups.

Genus	P-value	HR	HR (2.5 percentile)	HR (97.5 percentile)	% Group1	% Group0
D_0__Bacteria.D_1__Actinobacteria.D_2__Actinobacteria.D_3__Actinomycetales.D_4__Actinomycetaceae.D_5__Actinomyces	0,56	1,38	0,46	4,12	50	40
D_0__Bacteria.D_1__Actinobacteria.D_2__Actinobacteria.D_3__Micrococcales.D_4__Micrococcaceae.D_5__Rothia	0,23	0,51	0,17	1,55	36	53
D_0__Bacteria.D_1__Actinobacteria.D_2__Coriobacteriia.D_3__Coriobacteriales.D_4__Atopobiaceae.D_5__Atopobium	0,53	1,41	0,47	4,22	50	40
D_0__Bacteria.D_1__Bacteroidetes.D_2__Bacteroidia.D_3__Bacteroidales.D_4__Porphyromonadaceae.D_5__Porphyromonas	0,95	1,04	0,35	3,08	43	47
D_0__Bacteria.D_1__Bacteroidetes.D_2__Bacteroidia.D_3__Bacteroidales.D_4__Prevotellaceae.D_5__Alloprevotella	0,48	0,67	0,22	2,05	36	53
D_0__Bacteria.D_1__Bacteroidetes.D_2__Bacteroidia.D_3__Bacteroidales.D_4__Prevotellaceae.D_5__Prevotella	0,56	1,38	0,46	4,13	50	40
D_0__Bacteria.D_1__Bacteroidetes.D_2__Bacteroidia.D_3__Bacteroidales.D_4__Prevotellaceae.D_5__Prevotella.6	0,53	1,41	0,47	4,22	50	40
D_0__Bacteria.D_1__Bacteroidetes.D_2__Bacteroidia.D_3__Bacteroidales.D_4__Prevotellaceae.D_5__Prevotella.7	0,93	0,95	0,32	2,83	43	47
D_0__Bacteria.D_1__Bacteroidetes.D_2__Bacteroidia.D_3__Flavobacteriales.D_4__Flavobacteriaceae.D_5__Capnocytophaga	0,27	1,85	0,6	5,68	57	33
D_0__Bacteria.D_1__Bacteroidetes.D_2__Bacteroidia.D_3__Flavobacteriales.D_4__Weeksellaceae.D_5__Bergeyella	0,59	1,35	0,45	4,02	50	40
D_0__Bacteria.D_1__Epsilonbacteraeota.D_2__Campylobacteria.D_3__Campylobacterales.D_4__Campylobacteraceae.D_5__Campylobacter	0,46	1,51	0,51	4,5	50	40
D_0__Bacteria.D_1__Firmicutes.D_2__Bacilli.D_3__Bacillales.D_4__Family.XI.D_5__Gemella	0,27	0,54	0,18	1,64	36	53
D_0__Bacteria.D_1__Firmicutes.D_2__Bacilli.D_3__Lactobacillales.D_4__Carnobacteriaceae.D_5__Granulicatella	0,08	0,36	0,11	1,17	29	60
D_0__Bacteria.D_1__Firmicutes.D_2__Bacilli.D_3__Lactobacillales.D_4__Lactobacillaceae.D_5__Lactobacillus	0,21	2,01	0,65	6,16	57	33
D_0__Bacteria.D_1__Firmicutes.D_2__Bacilli.D_3__Lactobacillales.D_4__Streptococcaceae.D_5__Streptococcus	0,21	0,49	0,16	1,52	36	53
D_0__Bacteria.D_1__Firmicutes.D_2__Clostridia.D_3__Clostridiales.D_4__Lachnospiraceae.D_5__Catonella	0,74	1,2	0,4	3,58	50	40
D_0__Bacteria.D_1__Firmicutes.D_2__Clostridia.D_3__Clostridiales.D_4__Lachnospiraceae.D_5__Lachnoanaerobaculum	0,61	1,32	0,44	3,95	50	40
D_0__Bacteria.D_1__Firmicutes.D_2__Clostridia.D_3__Clostridiales.D_4__Lachnospiraceae.D_5__Oribacterium	0,69	1,25	0,42	3,72	50	40
D_0__Bacteria.D_1__Firmicutes.D_2__Clostridia.D_3__Clostridiales.D_4__Lachnospiraceae.D_5__Stomatobaculum	0,74	1,2	0,4	3,58	50	40
D_0__Bacteria.D_1__Firmicutes.D_2__Erysipelotrichia.D_3__Erysipelotrichales.D_4__Erysipelotrichaceae.D_5__Solobacterium	0,89	0,93	0,31	2,77	43	47
D_0__Bacteria.D_1__Firmicutes.D_2__Negativicutes.D_3__Selenomonadales.D_4__Veillonellaceae.D_5__Megasphaera	0,39	0,62	0,2	1,88	36	53
D_0__Bacteria.D_1__Firmicutes.D_2__Negativicutes.D_3__Selenomonadales.D_4__Veillonellaceae.D_5__Selenomonas.3	0,50	1,46	0,49	4,36	50	40
D_0__Bacteria.D_1__Firmicutes.D_2__Negativicutes.D_3__Selenomonadales.D_4__Veillonellaceae.D_5__Veillonella	0,55	1,4	0,47	4,16	50	40
D_0__Bacteria.D_1__Fusobacteria.D_2__Fusobacteriia.D_3__Fusobacteriales.D_4__Fusobacteriaceae.D_5__Fusobacterium	0,85	0,9	0,3	2,67	43	47
D_0__Bacteria.D_1__Fusobacteria.D_2__Fusobacteriia.D_3__Fusobacteriales.D_4__Leptotrichiaceae.D_5__Leptotrichia	0,86	0,91	0,31	2,71	43	47
D_0__Bacteria.D_1__Patescibacteria.D_2__Saccharimonadia.D_3__Saccharimonadales.D_4__Saccharimonadaceae.D_5__uncultured.bacterium	0,41	0,63	0,21	1,93	36	53
D_0__Bacteria.D_1__Proteobacteria.D_2__Gammaproteobacteria.D_3__Betaproteobacteriales.D_4__Neisseriaceae.D_5__Neisseria	0,30	0,56	0,18	1,7	36	53
D_0__Bacteria.D_1__Proteobacteria.D_2__Gammaproteobacteria.D_3__Pasteurellales.D_4__Pasteurellaceae.D_5__Haemophilus	0,42	0,63	0,21	1,94	36	53

Table S3: Association between genera relative abundance at preconditioning and oral mucositis (OM) risk. Groups were stratified based on median genus relative abundance and association with the risk of OM was assessed by estimating the Cox proportional hazards between groups. HR, Hazard Ratio; % Group1, percentage of patients classified as having high genus relative abundance at preconditioning that developed OM; % Group0, percentage of patients classified as having low genus relative abundance at preconditioning that developed OM.

Genus	P-value	Spearman rho
D_0__Bacteria.D_1__Actinobacteria.D_2__Actinobacteria.D_3__Actinomycetales.D_4__Actinomycetaceae.D_5__Actinomyces	0,57	0,11
D_0__Bacteria.D_1__Actinobacteria.D_2__Actinobacteria.D_3__Micrococcales.D_4__Micrococcaceae.D_5__Rothia	0,11	-0,30
D_0__Bacteria.D_1__Actinobacteria.D_2__Coriobacteriia.D_3__Coriobacteriales.D_4__Atopobiaceae.D_5__Atopobium	0,86	-0,03
D_0__Bacteria.D_1__Bacteroidetes.D_2__Bacteroidia.D_3__Bacteroidales.D_4__Porphyromonadaceae.D_5__Porphyromonas	0,96	0,01
D_0__Bacteria.D_1__Bacteroidetes.D_2__Bacteroidia.D_3__Bacteroidales.D_4__Prevotellaceae.D_5__Alloprevotella	0,78	0,05
D_0__Bacteria.D_1__Bacteroidetes.D_2__Bacteroidia.D_3__Bacteroidales.D_4__Prevotellaceae.D_5__Prevotella	0,15	0,27
D_0__Bacteria.D_1__Bacteroidetes.D_2__Bacteroidia.D_3__Bacteroidales.D_4__Prevotellaceae.D_5__Prevotella.6	0,45	0,15
D_0__Bacteria.D_1__Bacteroidetes.D_2__Bacteroidia.D_3__Bacteroidales.D_4__Prevotellaceae.D_5__Prevotella.7	0,82	-0,04
D_0__Bacteria.D_1__Bacteroidetes.D_2__Bacteroidia.D_3__Flavobacteriales.D_4__Flavobacteriaceae.D_5__Capnocytophaga	0,37	0,17
D_0__Bacteria.D_1__Bacteroidetes.D_2__Bacteroidia.D_3__Flavobacteriales.D_4__Weeksellaceae.D_5__Bergeyella	0,46	0,14
D_0__Bacteria.D_1__Epsilonbacteraeota.D_2__Campylobacteria.D_3__Campylobacterales.D_4__Campylobacteraceae.D_5__Campylobacter	0,95	-0,01
D_0__Bacteria.D_1__Firmicutes.D_2__Bacilli.D_3__Bacillales.D_4__Family.XI.D_5__Gemella	0,71	-0,07
D_0__Bacteria.D_1__Firmicutes.D_2__Bacilli.D_3__Lactobacillales.D_4__Carnobacteriaceae.D_5__Granulicatella	0,76	-0,06
D_0__Bacteria.D_1__Firmicutes.D_2__Bacilli.D_3__Lactobacillales.D_4__Lactobacillaceae.D_5__Lactobacillus	0,95	0,01
D_0__Bacteria.D_1__Firmicutes.D_2__Bacilli.D_3__Lactobacillales.D_4__Streptococcaceae.D_5__Streptococcus	0,06	-0,35
D_0__Bacteria.D_1__Firmicutes.D_2__Clostridia.D_3__Clostridiales.D_4__Lachnospiraceae.D_5__Catonella	0,64	0,09
D_0__Bacteria.D_1__Firmicutes.D_2__Clostridia.D_3__Clostridiales.D_4__Lachnospiraceae.D_5__Lachnoanaerobaculum	0,74	0,06
D_0__Bacteria.D_1__Firmicutes.D_2__Clostridia.D_3__Clostridiales.D_4__Lachnospiraceae.D_5__Oribacterium	0,61	0,10
D_0__Bacteria.D_1__Firmicutes.D_2__Clostridia.D_3__Clostridiales.D_4__Lachnospiraceae.D_5__Stomatobaculum	0,58	0,11
D_0__Bacteria.D_1__Firmicutes.D_2__Erysipelotrichia.D_3__Erysipelotrichales.D_4__Erysipelotrichaceae.D_5__Solobacterium	0,25	0,22
D_0__Bacteria.D_1__Firmicutes.D_2__Negativicutes.D_3__Selenomonadales.D_4__Veillonellaceae.D_5__Megasphaera	0,73	-0,07
D_0__Bacteria.D_1__Firmicutes.D_2__Negativicutes.D_3__Selenomonadales.D_4__Veillonellaceae.D_5__Selenomonas.3	0,90	-0,03
D_0__Bacteria.D_1__Firmicutes.D_2__Negativicutes.D_3__Selenomonadales.D_4__Veillonellaceae.D_5__Veillonella	0,79	0,05
D_0__Bacteria.D_1__Fusobacteria.D_2__Fusobacteriia.D_3__Fusobacteriales.D_4__Fusobacteriaceae.D_5__Fusobacterium	0,76	-0,06
D_0__Bacteria.D_1__Fusobacteria.D_2__Fusobacteriia.D_3__Fusobacteriales.D_4__Leptotrichiaceae.D_5__Leptotrichia	0,99	0,00
D_0__Bacteria.D_1__Patescibacteria.D_2__Saccharimonadia.D_3__Saccharimonadales.D_4__Saccharimonadaceae.D_5__uncultured.bacterium	0,94	0,02
D_0__Bacteria.D_1__Proteobacteria.D_2__Gammaproteobacteria.D_3__Betaproteobacteriales.D_4__Neisseriaceae.D_5__Neisseria	0,60	0,10
D_0__Bacteria.D_1__Proteobacteria.D_2__Gammaproteobacteria.D_3__Pasteurellales.D_4__Pasteurellaceae.D_5__Haemophilus	0,54	-0,12

Table S4: Correlation between genera relative abundance at preconditioning and ulcerative oral mucositis (OM) grade. All OM grades (0-4) are considered in this analysis. Spearman correlation was used.

Genus	P-value	Spearman rho
D_0__Bacteria.D_1__Actinobacteria.D_2__Actinobacteria.D_3__Actinomycetales.D_4__Actinomycetaceae.D_5__Actinomyces	0,69	0,12
D_0__Bacteria.D_1__Actinobacteria.D_2__Actinobacteria.D_3__Micrococcales.D_4__Micrococcaceae.D_5__Rothia	0,67	-0,13
D_0__Bacteria.D_1__Actinobacteria.D_2__Coriobacteriia.D_3__Coriobacteriales.D_4__Atopobiaceae.D_5__Atopobium	0,89	-0,04
D_0__Bacteria.D_1__Bacteroidetes.D_2__Bacteroidia.D_3__Bacteroidales.D_4__Porphyromonadaceae.D_5__Porphyromonas	0,03	0,61
D_0__Bacteria.D_1__Bacteroidetes.D_2__Bacteroidia.D_3__Bacteroidales.D_4__Prevotellaceae.D_5__Alloprevotella	0,07	0,51
D_0__Bacteria.D_1__Bacteroidetes.D_2__Bacteroidia.D_3__Bacteroidales.D_4__Prevotellaceae.D_5__Prevotella	0,59	0,17
D_0__Bacteria.D_1__Bacteroidetes.D_2__Bacteroidia.D_3__Bacteroidales.D_4__Prevotellaceae.D_5__Prevotella.6	0,66	-0,14
D_0__Bacteria.D_1__Bacteroidetes.D_2__Bacteroidia.D_3__Bacteroidales.D_4__Prevotellaceae.D_5__Prevotella.7	0,47	-0,22
D_0__Bacteria.D_1__Bacteroidetes.D_2__Bacteroidia.D_3__Flavobacteriales.D_4__Flavobacteriaceae.D_5__Capnocytophaga	0,46	0,22
D_0__Bacteria.D_1__Bacteroidetes.D_2__Bacteroidia.D_3__Flavobacteriales.D_4__Weeksellaceae.D_5__Bergeyella	0,22	0,37
D_0__Bacteria.D_1__Epsilonbacteraeota.D_2__Campylobacteria.D_3__Campylobacterales.D_4__Campylobacteraceae.D_5__Campylobacter	0,37	-0,27
D_0__Bacteria.D_1__Firmicutes.D_2__Bacilli.D_3__Bacillales.D_4__Family.XI.D_5__Gemella	0,82	-0,07
D_0__Bacteria.D_1__Firmicutes.D_2__Bacilli.D_3__Lactobacillales.D_4__Carnobacteriaceae.D_5__Granulicatella	0,44	0,23
D_0__Bacteria.D_1__Firmicutes.D_2__Bacilli.D_3__Lactobacillales.D_4__Lactobacillaceae.D_5__Lactobacillus	0,31	-0,31
D_0__Bacteria.D_1__Firmicutes.D_2__Bacilli.D_3__Lactobacillales.D_4__Streptococcaceae.D_5__Streptococcus	0,52	-0,20
D_0__Bacteria.D_1__Firmicutes.D_2__Clostridia.D_3__Clostridiales.D_4__Lachnospiraceae.D_5__Catonella	0,65	-0,14
D_0__Bacteria.D_1__Firmicutes.D_2__Clostridia.D_3__Clostridiales.D_4__Lachnospiraceae.D_5__Lachnoanaerobaculum	0,40	-0,26
D_0__Bacteria.D_1__Firmicutes.D_2__Clostridia.D_3__Clostridiales.D_4__Lachnospiraceae.D_5__Oribacterium	0,47	0,22
D_0__Bacteria.D_1__Firmicutes.D_2__Clostridia.D_3__Clostridiales.D_4__Lachnospiraceae.D_5__Stomatobaculum	0,67	0,13
D_0__Bacteria.D_1__Firmicutes.D_2__Erysipelotrichia.D_3__Erysipelotrichales.D_4__Erysipelotrichaceae.D_5__Solobacterium	0,35	0,29
D_0__Bacteria.D_1__Firmicutes.D_2__Negativicutes.D_3__Selenomonadales.D_4__Veillonellaceae.D_5__Megasphaera	0,26	-0,34
D_0__Bacteria.D_1__Firmicutes.D_2__Negativicutes.D_3__Selenomonadales.D_4__Veillonellaceae.D_5__Selenomonas.3	0,20	-0,38
D_0__Bacteria.D_1__Firmicutes.D_2__Negativicutes.D_3__Selenomonadales.D_4__Veillonellaceae.D_5__Veillonella	0,21	-0,37
D_0__Bacteria.D_1__Fusobacteria.D_2__Fusobacteriia.D_3__Fusobacteriales.D_4__Fusobacteriaceae.D_5__Fusobacterium	0,67	0,13
D_0__Bacteria.D_1__Fusobacteria.D_2__Fusobacteriia.D_3__Fusobacteriales.D_4__Leptotrichiaceae.D_5__Leptotrichia	0,51	-0,20
D_0__Bacteria.D_1__Patescibacteria.D_2__Saccharimonadia.D_3__Saccharimonadales.D_4__Saccharimonadaceae.D_5__uncultured.bacterium	0,95	-0,02
D_0__Bacteria.D_1__Proteobacteria.D_2__Gammaproteobacteria.D_3__Betaproteobacteriales.D_4__Neisseriaceae.D_5__Neisseria	0,19	0,39
D_0__Bacteria.D_1__Proteobacteria.D_2__Gammaproteobacteria.D_3__Pasteurellales.D_4__Pasteurellaceae.D_5__Haemophilus	0,23	-0,35

Table S5: Correlation between genera relative abundance at preconditioning and ulcerative oral mucositis (OM) grade. Only ulcerative OM grades (2-4) are considered in this analysis. Spearman correlation was used.

Genus	P-value	HR	HR (2.5 percentile)	HR (97.5 percentile)
D_0__Bacteria.D_1__Actinobacteria.D_2__Actinobacteria.D_3__Actinomycetales.D_4__Actinomycetaceae.D_5__Actinomyces	0,42	1,59	0,51	5,01
D_0__Bacteria.D_1__Actinobacteria.D_2__Actinobacteria.D_3__Bifidobacteriales.D_4__Bifidobacteriaceae.__	0,16	2,47	0,67	9,01
D_0__Bacteria.D_1__Actinobacteria.D_2__Actinobacteria.D_3__Micrococcales.D_4__Micrococcaceae.D_5__Rothia	0,94	1,04	0,33	3,34
D_0__Bacteria.D_1__Actinobacteria.D_2__Coriobacteriia.D_3__Coriobacteriales.D_4__Atopobiaceae.D_5__Atopobium	0,95	1,04	0,33	3,25
D_0__Bacteria.D_1__Bacteroidetes.D_2__Bacteroidia.D_3__Bacteroidales.D_4__Porphyromonadaceae.D_5__Porphyromonas	0,07	0,3	0,08	1,2
D_0__Bacteria.D_1__Bacteroidetes.D_2__Bacteroidia.D_3__Bacteroidales.D_4__Prevotellaceae.D_5__Alloprevotella	0,86	1,1	0,35	3,45
D_0__Bacteria.D_1__Bacteroidetes.D_2__Bacteroidia.D_3__Bacteroidales.D_4__Prevotellaceae.D_5__Prevotella	0,88	1,09	0,35	3,43
D_0__Bacteria.D_1__Bacteroidetes.D_2__Bacteroidia.D_3__Bacteroidales.D_4__Prevotellaceae.D_5__Prevotella.6	0,28	2,03	0,54	7,55
D_0__Bacteria.D_1__Bacteroidetes.D_2__Bacteroidia.D_3__Bacteroidales.D_4__Prevotellaceae.D_5__Prevotella.7	0,77	0,84	0,27	2,66
D_0__Bacteria.D_1__Bacteroidetes.D_2__Bacteroidia.D_3__Flavobacteriales.D_4__Flavobacteriaceae.D_5__Capnocytophaga	0,84	0,89	0,28	2,81
D_0__Bacteria.D_1__Epsilonbacteraeota.D_2__Campylobacteria.D_3__Campylobacterales.D_4__Campylobacteraceae.D_5__Campylobacter	0,98	0,99	0,32	3,08
D_0__Bacteria.D_1__Firmicutes.D_2__Bacilli.D_3__Bacillales.D_4__Family.XI.D_5__Gemella	0,08	0,3	0,08	1,24
D_0__Bacteria.D_1__Firmicutes.D_2__Bacilli.D_3__Lactobacillales.D_4__Carnobacteriaceae.D_5__Granulicatella	0,96	1,03	0,33	3,23
D_0__Bacteria.D_1__Firmicutes.D_2__Bacilli.D_3__Lactobacillales.D_4__Lactobacillaceae.D_5__Lactobacillus	0,04	4,99	0,96	26,04
D_0__Bacteria.D_1__Firmicutes.D_2__Bacilli.D_3__Lactobacillales.D_4__Streptococcaceae.D_5__Streptococcus	0,15	0,4	0,11	1,42
D_0__Bacteria.D_1__Firmicutes.D_2__Clostridia.D_3__Clostridiales.D_4__Lachnospiraceae.D_5__Lachnoanaerobaculum	0,16	0,38	0,1	1,51
D_0__Bacteria.D_1__Firmicutes.D_2__Clostridia.D_3__Clostridiales.D_4__Lachnospiraceae.D_5__Oribacterium	0,91	1,07	0,34	3,35
D_0__Bacteria.D_1__Firmicutes.D_2__Negativicutes.D_3__Selenomonadales.D_4__Veillonellaceae.D_5__Selenomonas.3	0,16	2,47	0,67	9,01
D_0__Bacteria.D_1__Firmicutes.D_2__Negativicutes.D_3__Selenomonadales.D_4__Veillonellaceae.D_5__Veillonella	0,10	0,33	0,08	1,3
D_0__Bacteria.D_1__Fusobacteria.D_2__Fusobacteriia.D_3__Fusobacteriales.D_4__Fusobacteriaceae.D_5__Fusobacterium	0,39	0,6	0,19	1,92
D_0__Bacteria.D_1__Fusobacteria.D_2__Fusobacteriia.D_3__Fusobacteriales.D_4__Leptotrichiaceae.D_5__Leptotrichia	0,97	1,02	0,33	3,2

Table S6: Association between genera relative abundance at oral mucositis (OM) onset and time to OM healing. Groups were stratified based on median genus relative abundance and association with time to OM healing was assessed by estimating the Cox proportional hazards between groups. HR, Hazard Ratio.
